# Azithromycin ameliorates sulfur dioxide-induced airway epithelial damage and inflammatory responses

**DOI:** 10.1186/s12931-020-01489-8

**Published:** 2020-09-10

**Authors:** Jon Petur Joelsson, Jennifer A. Kricker, Ari J. Arason, Snaevar Sigurdsson, Bryndis Valdimarsdottir, Fridrik Runar Gardarsson, Clive P. Page, Fredrik Lehmann, Thorarinn Gudjonsson, Saevar Ingthorsson

**Affiliations:** 1 Stem Cell Research Unit, BioMedical Center, School of Health Sciences, University of Iceland, Reykjavík, Iceland; 2 EpiEndo Pharmaceuticals, Reykjavík, Iceland; 3 Department of Laboratory Hematology, Landspitali-University Hospital, Reykjavík, Iceland; 4 Biomedical Center, University of Iceland, Reykjavík, Iceland; 5 Sackler Institute of Pulmonary Pharmacology, King’s College London, London, UK; 6 Faculty of Nursing, University of Iceland, Reykjavík, Iceland

**Keywords:** Azithromycin, Immunomodulation, Lung barrier enhancement, Glutathione-S-transferase, Lamellar bodies

## Abstract

**Background:**

The airway epithelium (AE) forms the first line of defence against harmful particles and pathogens. Barrier failure of the airway epithelium contributes to exacerbations of a range of lung diseases that are commonly treated with Azithromycin (AZM). In addition to its anti-bacterial function, AZM has immunomodulatory effects which are proposed to contribute to its clinical effectiveness. In vitro studies have shown the AE barrier-enhancing effects of AZM. The aim of this study was to analyze whether AE damage caused by inhalation of sulfur dioxide (SO_2_) in a murine model could be reduced by pre-treatment with AZM.

**Methods:**

The leakiness of the AE barrier was evaluated after SO_2_ exposure by measuring levels of human serum albumin (HSA) in bronchoalveolar lavage fluid (BALF). Protein composition in BALF was also assessed and lung tissues were evaluated across treatments using histology and gene expression analysis.

**Results:**

AZM pre-treatment (2 mg/kg p.o. 5 times/week for 2 weeks) resulted in reduced glutathione-S-transferases in BALF of SO_2_ injured mice compared to control (without AZM treatment). AZM treated mice had increased intracellular vacuolization including lamellar bodies and a reduction in epithelial shedding after injury in addition to a dampened SO_2_-induced inflammatory response.

**Conclusions:**

Using a mouse model of AE barrier dysfunction we provide evidence for the protective effects of AZM in vivo, possibly through stabilizing the intracellular microenvironment and reducing inflammatory responses. Our data provide insight into the mechanisms contributing to the efficacy of AZM in the treatment of airway diseases.

## Background

Environmental pollutants are often associated with exacerbation of disease, including those linked to cardiac, skin and respiratory conditions [1, 2]. Air pollution is comprised of several components, one of the major ones being sulfur dioxide (SO_2_), a gas commonly produced from combustion of sulfur-containing fuels such as coal as well as from volcanic eruptions [3]. Fine particles formed from fugitive dust, SO_2_ and oxygen result in particulate matter (PM), a primary pollutant and irritant which, as air pollution increases, leads to an increase in respiratory and cardiovascular diseases [1].

The lungs, lined with an epithelial layer, are complex structures responsible for filtering and humidifying air, facilitating gas exchange, and acting as the first line of defense against the external environment [4]. The airway epithelium from the nose, trachea and bronchi through to the alveoli, together with the underlying basement membrane form an effective barrier to prevent pollutants and infectious agents such as bacteria and viruses from entering the body [5]. Exposure to air pollutants, both gaseous and PM, can weaken the epithelial barrier, predispose the respiratory system to infections, and facilitate both acute and chronic respiratory disorders [6]. Indeed, fine PM have been shown to lead to a weakened epithelium [7, 8] and are now accepted as a leading contributor to chronic respiratory and cardiovascular diseases [9, 10].

Patients suffering from asthma, COPD and CF are at greatest risk of exacerbations caused by air pollution such as SO_2_ and PMs, along with viruses and bacteria. These patients often have compromised lung barrier functions [6]. Treatment of patients with acute exacerbations of respiratory diseases often involves administration of antibiotics such as macrolides including AZM [11]. Indeed, a range of studies have revealed beneficial effects of AZM beyond its anti-microbial effects that has led to this macrolide being used for chronic maintenance treatment to prevent exacerbations of respiratory diseases, a treatment approach that whilst effective, increases the risk of bacterial resistance [11]. AZM has been shown to have multiple disease-modifying effects of relevance to treating and preventing exacerbations of respiratory diseases, including immunomodulation (reviewed by Parnham et al. [12]).

In addition to the anti-microbial and immunomodulatory effects of AZM, previous studies by our group and others have shown that this drug enhances the respiratory epithelial barrier when cultured in air-liquid interface (ALI) culture [13–15]. Most recently, we demonstrated that AZM induces a partial epidermal differentiation program in bronchial epithelial cells in ALI culture [16]. Histological analysis demonstrated formation of lamellar bodies (LBs). LBs are found in lungs, where they contribute to production and release of pulmonary surfactants, and in skin, where they contribute to the water barrier, presumably facilitating the increased barrier function observed in vitro. However, to date there have been no observations demonstrating similar barrier enhancing effects of AZM in vivo. In this study, we have established an in vivo model of epithelial barrier dysfunction in the airway epithelium using SO_2_ exposure to investigate whether AZM has barrier protective functions. We demonstrate that pretreating mice with AZM prior to SO_2_ exposure ameliorates AE damage including barrier leakiness, reduces the expression of GST detoxification enzymes and dampens the interferon–alpha associated inflammatory responses.

## Materials and methods

### Mice

Female C57BL/6NTac mice were purchased from Taconic Biosciences, Denmark. Mice were purchased within the age range of 5–9 weeks, with an average weight of 20 g on arrival. Mice were allowed 1 week of acclimatization before the start of the study. They were randomized into treatment groups (6 mice in a group per cage) and kept in 335 cm^2^ cages 13 cm deep. Cages were housed in standardized environments in Scanbur cabinets. The animals were maintained with a 12-h light cycle at 23–24 °C and relative air humidity of 40% Mice were kept on aspen bedding with nesting material, red polycarbonate hiding material and biting bricks. They were provided with ad libitum Altromin diet and filtered drinking water. This study has been approved by the Icelandic food and veterinary authority (MAST), Licence #2018-09-02. The choice of using 6 mice per group was based on a similar study [17].

### AZM treatment

AZM was provided by Recipharm, Uppsala, Sweden. Mice (*n* = 6 in each group) were treated p.o. with 2 mg/kg AZM in 5% ethanol in saline 5 times per week for 2 weeks prior to SO_2_ exposure. Control animals (*n* = 6 in each group) received 5% ethanol in saline for the same duration.

### SO_2_ exposure

Inhalation exposure was regulated using a system from Electro-Medical Measurement Systems (EMMS, UK). SO_2_ was purchased as 500 ppm in 80%/20% N_2_/O_2_ (synthair) from ÍSAGA (Reykjavik, Iceland). The inhalation system was custom-built to facilitate controlled exposure of individual mice. Mice were placed in individual holding tubes and placed in a holding tower so that their noses were inside the tower for inhalation. SO_2_ was downmixed in air by the system to the desired concentration and pumped into the tower at the top at a rate of 5 L/min. Tower pressure was maintained at a positive pressure of 0.3 cm H_2_O by active monitoring and active outflow (vacuum pump). The SO_2_ was bubbled through concentrated NaOH in water for scrubbing.

Mice were exposed to 50–400 ppm SO_2_ for 4 h. After SO_2_ exposure, mice were given 1–7 days to recover before BALF collection and subsequent histological and molecular analysis of lung tissue. Animal weight was also monitored before and after exposure to SO_2_ gas.

### BALF collection

BALF was collected 1, 3 or 7 days after SO_2_ exposure. Sixty minutes before BALF collection, mice were injected i.v. via the tail vein with 1 mg HSA in a 100 μl bolus of saline. Before BALF collection, mice were euthanized with a mixture of Euthasol vet/Lidocaine. After euthanasia, mice were placed in a supine position and the trachea exposed by cutting the skin and moving the salivary glands. Using sharp forceps, a suture thread was inserted below the trachea. A small opening was cut in the trachea and the trachea intubated with a canula. The suture was then tied to hold the canula in place. BALF was collected from two 0.5 ml PBS washes and centrifuged at 2000 rpm for 3 min to collect pellet debris. BALF, free of cellular debris, was divided into three aliquots and stored at − 20 °C for future analyses.

### ELISA

HSA concentrations in BALF samples were measured by ELISA (R&D Systems, DY1455) according to the manufacturer’s instructions. In short, plates were coated with diluted anti-HSA antibodies overnight at room temperature (RT) and then blocked with blocking solution for 1 h at RT. Standards and samples were then added, incubated, and washed 4x with washing solution. Detection antibodies diluted in ELISA buffer were then added and incubated for 2 h at RT. Streptavidin-HRP (1:40 in ELISA buffer) was added and incubated in the dark at RT for 20 min. Substrate solution was then added and incubated in the dark at RT until colored precipitates were evident in most concentrated standards. Then stop solution was added and plates measured.

### Immunohistochemistry

After BALF collection, lungs were harvested for histology. Lungs were fixed in 3.7% buffered formaldehyde for 24 h minimum and embedded in paraffin using standardized procedures. After embedding, slides were prepared and stained with hematoxylin and eosin for assessment of histopathological effects.

### Transmission electron microscopy

Tracheas and lung pieces were fixed in 2.5% glutaraldehyde for 1–2 h followed by post-fixation in 2% osmium tetroxide for 1 h and a subsequent phosphate buffer rinse. Samples were dehydrated in ethanol and uranyl acetate and embedded in resin. 100 nm sections were cut with an Ultramicrotome (Leica EM UC7). Sections were stained with lead citrate (3%, J.T. Baker Chemical Co.) and imaged using a JEM-1400PLUS PL Transmission Electron Microscope.

### Proteomic analysis of BALF samples

BALF was collected 3 days after exposure and pooled from each animal (*n* = 6) in the separate treatment groups and concentrated (Amicon Ultra-15 filter units). Briefly, approximately 900 μl of pooled BALF was transferred to a 3 kDa Amicon column and spun as per manufacturer’s instructions. 100 μl of ~ 0.4 mg/ml BALF samples for each treatment group (*n* = 1) were for sent for LFQ analysis by MS to the “FingerPrints” Proteomic Facility, University of Dundee, UK.

### RNA isolation and RNA sequencing

Three days after exposure, mice were sacrificed and tissue pieces from lung were harvested and stored in RNAlater™ (Invitrogen, ThermoFisher) at − 20 °C until total RNA was extracted in TRI-Reagent® (Ambion, ThermoFisher) using gentle MACS™ Dissociator from Miltenyi Biotec in M tubes (Miltenyi Biotec). RNA samples were shipped to BGI Genomics (Ole Maaloes Vej 3, 2200 København, Denmark) for RNA sequencing. The RNA transcript expression was quantified with Kallisto version 0.46.1 [18] using the Ensembl *Mus musculus* GRCm38 reference transcriptome [19]. Gene expression estimates were computed with the sleuth R package v0.30 [20]. Two samples were discarded because of inadequate quality (one placebo control and one placebo SO_2_ treated sample). We measured transcripts that were differentially expressed using Wald test in sleuth, in placebo SO_2_-treated mice (*n* = 2) vs control untreated mice (*n* = 2), compared to AZM- and (No SO_2_ exposure, *n* = 3) SO_2_-treated mice (*n* = 3). Gene set enrichment analysis was performed with the GSEA software v4.0.1 [21, 22]. Pre-ranked gene lists were prepared by ordering the genes (one transcript per gene) by expression difference significance (q-value multiplied with the sign of the log-fold change) and tested for enrichment in the MSigDB hallmark gene set collection [23].

### Statistical analysis

For determining statistical significance for mouse weight and ELISA analysis, a student t-test was utilized and calculated using GraphPad Prism 8.0. Error bars represent the standard deviation of the means. Calculations for the statistical significance for the differential gene expression were calculated using Wald test in sleuth.

## Results

### Set up and validation of the SO_2_ exposure system

**Fig. 1:**
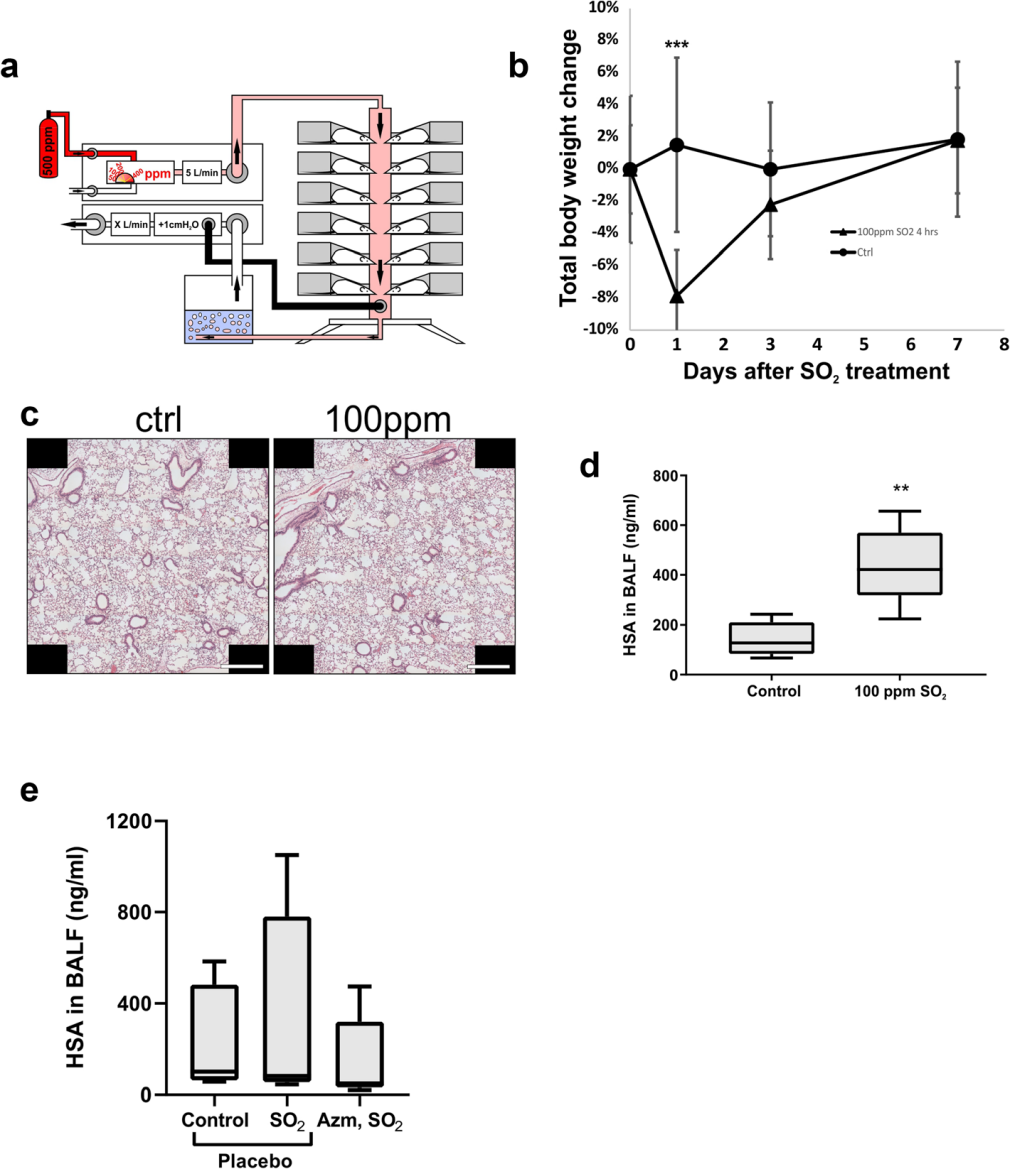
Subtle damage to airway epithelium and increased leakiness occurs after SO_2_ exposure which is mitigated by pretreatment with AZM. **a**. *Schematic depiction of the SO*_*2*_ *exposure system.* 500 ppm SO_2_ is downmixed to the desired concentration in a positive pressure control unit. Downmixed gas is passed over the animals in a tower, ensuring constant and comparable exposure to all animals. Up to 12 animals were treated simultaneously. Outflow gas was passed through a NaOH solution for scrubbing SO_2_ gas from the air mixture. Outflow pressure was monitored in order to maintain a small positive pressure (1 cm H_2_O) in the exposure tower. **b**. *Weight changes in mice exposed to 100 ppm SO*_*2*_ *for 4 h.* Overall, mice placed in the tower lost a significant (8%; *** *p* < 0.0001) amount of body weight after exposure to SO_2_ in the inhalation system. This weight loss did not occur in control animals. Weight loss was recovered quickly, and mice reached their starting weights within a week after exposure. Data are means of ± SD of *n* = 18 for SO_2_ exposed mice and *n* = 4 for control mice. **c**. *No histological changes were observed after exposure to SO*_*2*_ H&E stained lung tissue. No histological changes were observed. Scale bars are 500 μm. **d**. *HSA in BALF is increased upon SO*_*2*_ *exposure.* Increased HSA in SO_2_ exposed mice compared with control mice was seen, as determined by ELISA. Average HSA in control and SO_2_ treated mice was 143 and 440 ng/ml, respectively. Data are represented as box and whiskers plots with boxes encompassing the 25th – 75th quartiles, and whiskers demonstrating minimum and maximum values. *n* = 5 mice in each group. *p* = 0.0044. **e**. *AZM pre-treatment results in decreased epithelial permeability to HSA.* Mice orally gavaged with 2 mg/kg AZM 5 times per week had a trend for reduced HSA concentration in BALF observed after SO_2_ exposure when compared to placebo mice, indicating a protective effect. Average HSA in control, SO_2_ and AZM, SO_2_ groups was 238, 350 and 150 ng/ml, respectively. Data are represented as box and whiskers plots with boxes encompassing the 25th – 75th quartiles, and whiskers minimum and maximum values. *n* = 5 observations per treatment group. *p* = 0.13

Inhalation of SO_2_ was controlled using a custom-built inhalation system as described in the materials and methods. Figure 1a shows a diagram of the inhalation system to expose all animals in a comparable manner, while simultaneously preventing SO_2_ leakage into the environment. The whole system was kept in a fume hood, except for the input air- and SO_2_ containers. Mice were individually placed in size appropriate inhalation tubes and mounted to the tower for the duration of the experiment. Laboratory environmental heating was maintained at 26 °C to minimize the risk of hypothermia to the mice. After exposure, mice were returned to their cages to recover. Behaviorally, mice briefly demonstrated slight signs of distress, for example, hunching in corners of cages or hiding. Low mobility and lethargy were observed for about 60 min after exposure, accompanied by grooming. Afterwards, behavior returned to normal. On average, mice exposed to SO_2_ lost 8% of their body weight in the first 24 h after exposure, compared with no change in mice that were not exposed. Mice placed in the exposure system, but not exposed to SO_2_ for 4 h lost on average 3.5% body weight. Weight mostly recovered within 3 days and was fully recovered after 7 days (Fig. 1b).

### SO_2_ exposure causes no obvious histological changes, but increased airway epithelial permeability which can be reduced by AZM pre-treatment

Figure 1c, top row, demonstrates hematoxylin and eosin staining of histological sections of lung tissues exposed for 4 h to 100 ppm SO_2_, 1 day after exposure. No obvious histological changes were observed, and lungs appeared healthy.

One day after SO_2_ challenge, HSA in BALF was measured by ELISA and a significant (*p* = 0.004, *n* = 5 mice in each group) increase in HSA in BALF was observed in mice exposed to 100 ppm SO_2_ for 4 h (Fig. 1d), demonstrating an increased leakiness of the lungs, despite the apparent lack of visual damage, indicating sub-histological changes. When we compared HSA levels and histology in mice that were allowed 1-, 3- or 7-days recovery time, differences were minimal, therefore 3 days post SO_2_ exposure was selected for analyses for the remainder of the study. We hypothesized that 3 days would allow partial repair and we could detect the beneficial effects of pre-treatment with AZM. We pre-treated mice with 2 mg/kg AZM, dosed 5 times per week via oral gavage for 2 weeks. We then exposed these mice to 100 ppm SO_2_ for 4 h followed by a 3-day recovery time before BALF collection. HSA ELISA comparing SO_2_ exposed mice with SO_2_ exposed mice that received AZM indicated a trend (*p* = 0.13, *n* = 5 mice in each group) that the leakiness was attenuated as demonstrated by reduced HSA concentrations in BALF in the AZM treated group (Fig. 1e).

### SO_2_ exposure results in increased glutathione-S-transferase response that is reduced in AZM pre-treated mice

**Fig. 2:**
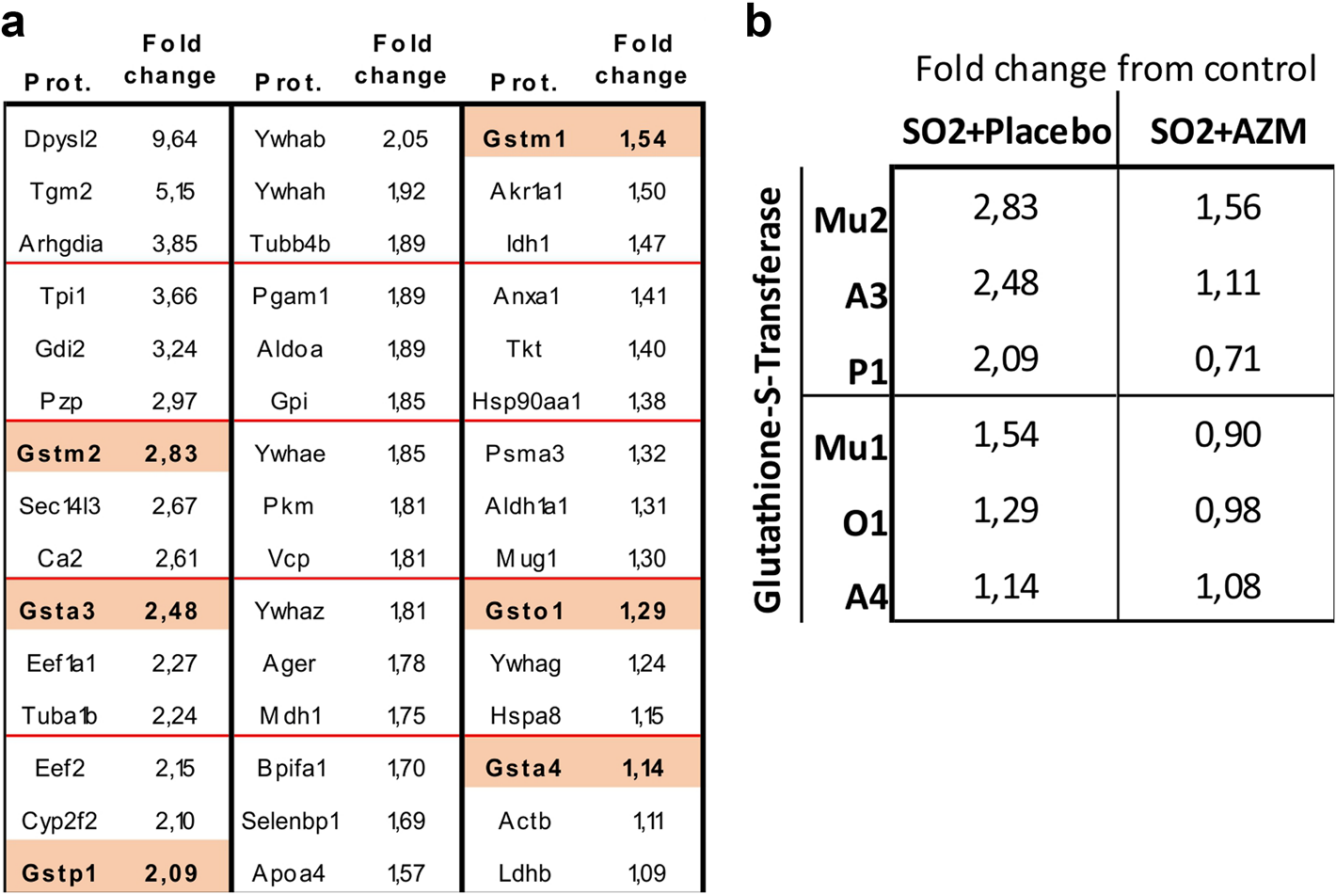
SO_2_ exposure results in increased expression of members of the Glutathione S Transferase family. **a**. *Glutathione S transferase is increased in BALF in SO*_*2*_ *exposed mice.* BALF samples were analysed by mass spectrometry. Of the samples showing dramatic changes from controls, 6 GST analogs were detected and demonstrated to be increased in BALF samples of SO_2_ exposed mice. Data represent a single measurement from a pooled sample from six mice in each treatment group. **b**. *The increased GST concentration in BALF seen in SO*_*2*_ *exposed mice is attenuated by pre-treatment with AZM.* The six GSTs shown previously all had reduced BALF concentrations when mice were pre-treated with AZM. Data represent a single measurement from a pooled sample from six mice in each treatment group

To extend the observations of epithelial permeability assessed by HSA measurements, we performed proteomic mass spectrometry (MS) of BALF to assess proteins and peptides in SO_2_ exposed mice compared to unexposed mice. We probed the dataset for proteins and peptides that were present in greater amounts in BALF from mice exposed to SO_2_ and discovered that 6 members of the glutathione-S-transferase (GST) superfamily were shown to be increased (Fig. 2a).

Interestingly, the GST family of proteins that showed increased expression in Fig. 2a were all considerably less expressed in mice pre-treated with AZM (Fig. 2b).

### Gene set enrichment analysis reveals attenuated inflammatory response to SO_2_ challenge as a result of AZM pre-treatment

**Fig. 3:**
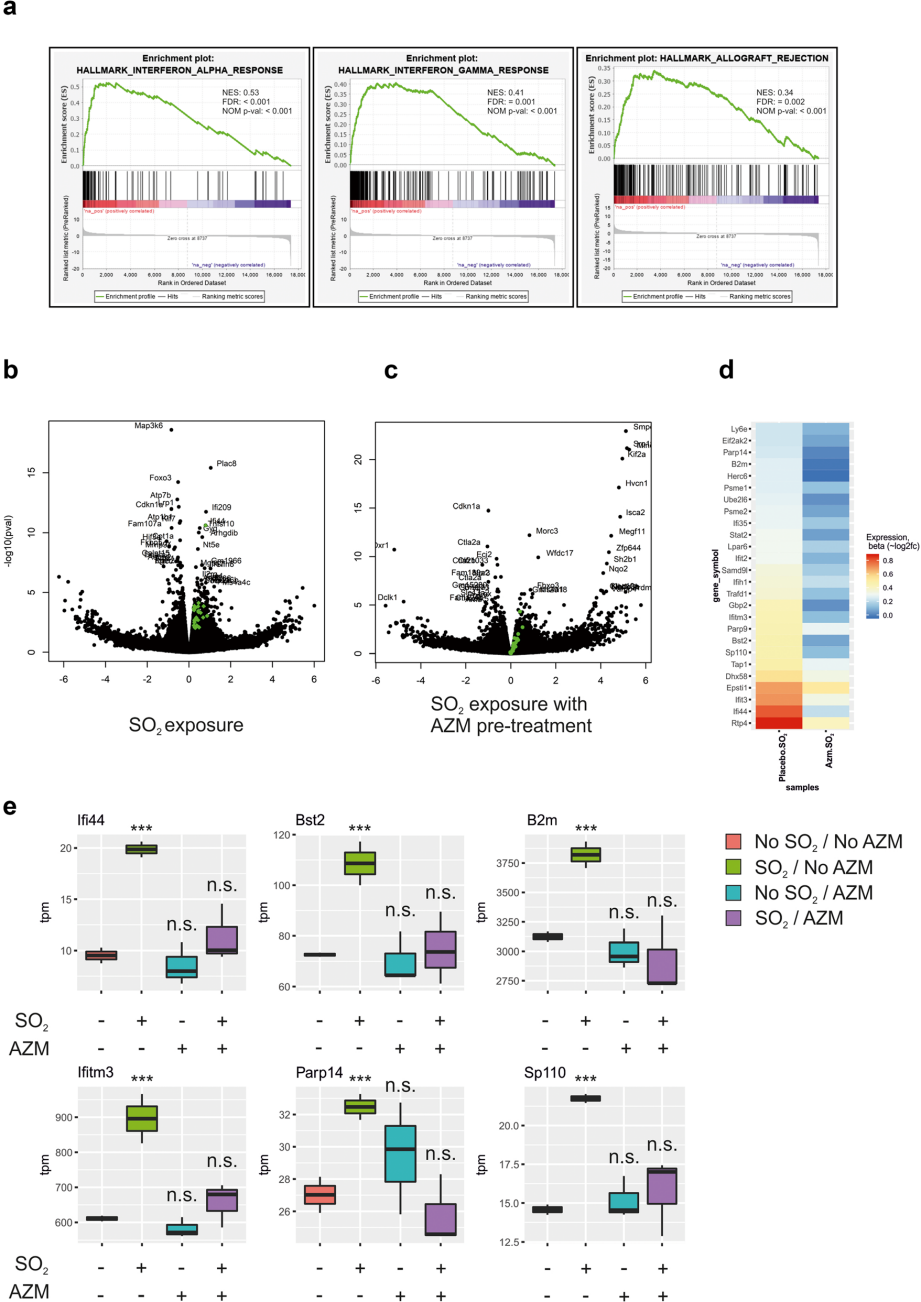
Pretreatment with AZM reduces the expression of SO_2_ induced inflammatory interferon alpha responsive genes. **a**. *Gene set enrichment analysis of positively correlated pathways with the highest normalized enrichment scores (NES) as a result of SO*_*2*_ *challenging of the mice.* Top three most positively correlated pathways were the inflammation related interferon alpha response, interferon gamma response and allograft rejection. FDR is false discovery rate and NOM p-val is nominal *p*-value. **b**. *Volcano plot of differentially expressed genes comparing gene expression of non-exposed* vs *SO*_*2*_ *exposed mice*. Genes in the interferon alpha response pathway are colored green. **c**. *Volcano plot of differentially expressed genes comparing gene expression of non-exposed* vs *SO*_*2*_ *exposed mice that had been pre-treated with AZM.* Genes in the interferon alpha response pathway are colored green. **d**. *Heatmap of genes that were positively correlated to the interferon alpha gene set.* On the left is shown the differential gene expression as a result of SO_2_ exposure. On the right is shown the differential expression as result of SO_2_ exposure in AZM pre-treated mice. **e**. *Boxplot of selected genes in the interferon alpha gene set.* All genes show a similar pattern of higher expression as a result of SO_2_ exposure. The AZM pre-treatment of the unexposed as well as the SO_2_ exposed resulted in similar expression levels as the unexposed, untreated mice. Boxplot made with the R package ggplot/geom. Data are represented as box and whiskers plots with boxes encompassing the 25th – 75th quartiles, and whiskers demonstrating 1.5 Interquartile range above and below. *N* = 3, except for placebo (*N* = 2). *** *p* ≤ 0.001

Whole lung RNA lysates were prepared and RNA transcript expression analysed via RNA sequencing. Analyzing the RNA sequencing data through gene set enrichment analysis (GSEA) showed that exposing the mice to SO_2_ resulted in a positive correlation with inflammatory gene pathways. The three pathways with the highest positively correlated normalized enrichment scores (NES) were interferon alpha response (NES: 0.53, p-val <0.001), interferon gamma response (NES: 0.41, p-val <0.001) and allograft rejection (NES: 0.34, <0.001) gene sets (Fig. 3a), all immune related pathways. The volcano plot presented in Fig. 3b shows the genes overrepresented in the gene set with the highest NES, interferon alpha response, in green. The volcano plot of the data obtained from SO_2_ exposed mice that had been pre-treated with AZM, shows a lower expression of these genes (Fig. 3c). Figure 3d presents a heatmap comparison of the interferon alpha genes involved. Analysis of several genes from the interferon alpha gene presents a similar pattern, i.e. that exposure to SO_2_ is followed by a significant (*p* ≤ 0.001) upregulation of the gene, but when mice were pre-treated with AZM, the gene expression stays closer to the levels observed in unexposed, untreated conditions and no significant difference is seen (Fig. 3e).

### AZM increases vesicle and lamellar body formations in both SO_2_ exposed and non-exposed mice

**Fig. 4:**
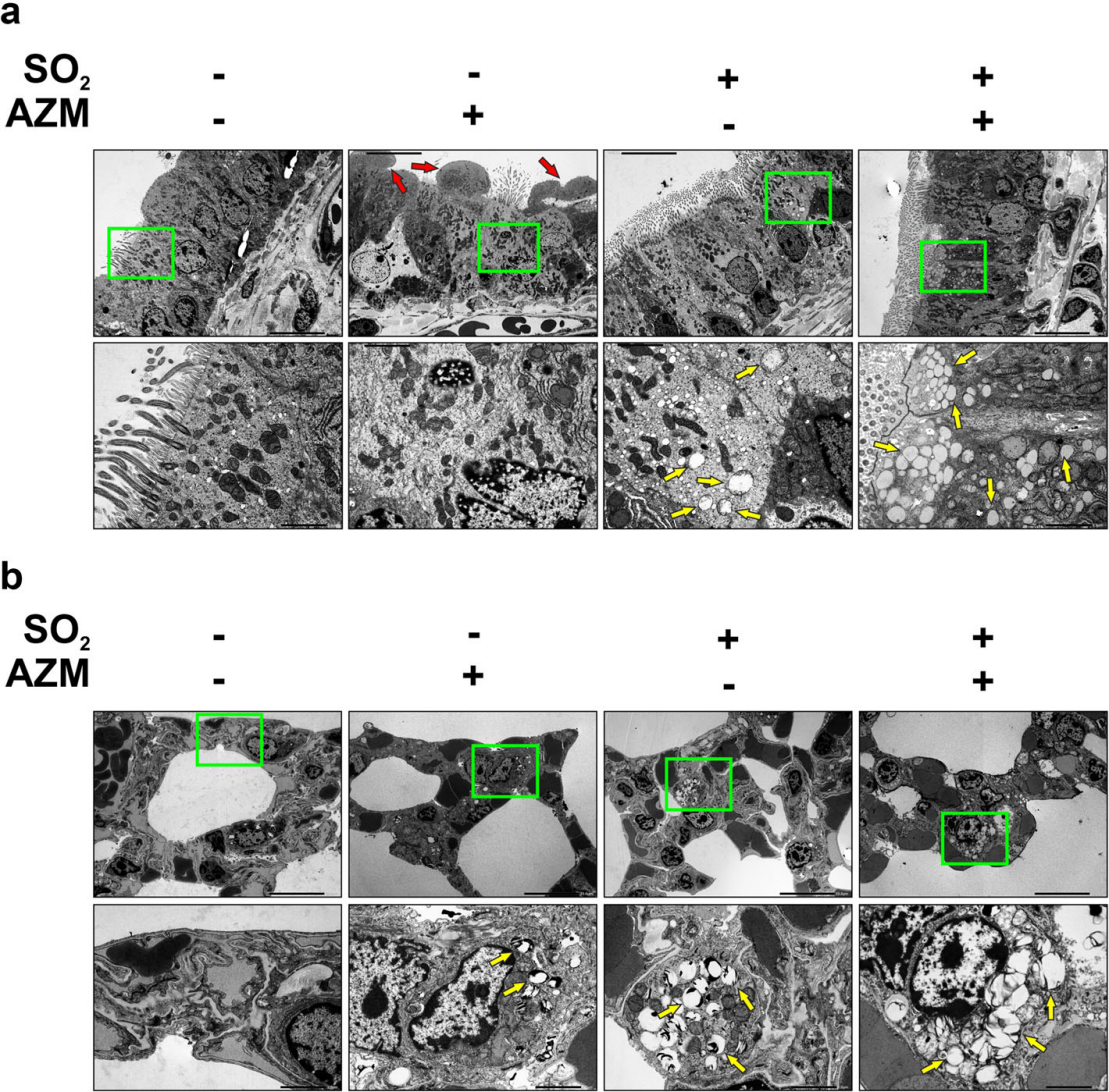
AZM pre-treatment protects against epithelial shedding and increases lamellar body formation. **a**. *Electron microscope images of the tracheal epithelium.* SO_2_ exposed mice show shedding of the epithelium as seen when comparing SO_2_ +/−. Shedding is seen by the top of the layer peeling of and an uneven layer indicated by red arrows. Mice that had been pre-treated with AZM, but not exposed to SO_2_ showed accumulations of vesicles in the epithelial layer. SO_2_ exposed, AZM pre-treated mice also showed these vesicle formations, even to a greater extent, and the epithelial layer did not show shedding. All the epithelial layers presented some small vesicle formations, but the increases as a result of AZM treatment were distinct throughout all the samples. Yellow arrows point to examples of vesicle formations. Shown are representative images taken of 3 replicates. Top scale bars are 10 μm and lower scale bars are 2 μm. **b**. *Electron microscope images of the alveolar region of the lungs.* No discernible difference was seen in the control vs the SO_2_ exposed mice. Lamellar bodies were observed in most of the alveolar type II cells in all the samples. Only in the AZM pre-treated mouse samples were accumulations of lamellar bodies detected. Yellow arrows point to examples of lamellar bodies. Shown are representative images taken of 3 replicates. Top scale bars are 10 μm and lower scale bars are 2 μm

Tracheal and alveolar tissue samples from the mice were collected 3 days after exposure. The tracheal epithelium of the SO_2_ exposed mice showed some signs of shedding, evidenced by unevenness of the epithelial layer and cells appearing to peel off from the top of the layer (Fig. 4a red arrows). When viewing tissues from AZM treated mice, there were accumulations of vesicles not seen in the same amounts as in the untreated controls (Fig. 4a yellow arrows). SO_2_ exposed mice that had received AZM pre-treatment accumulated even more vesicles and the layer was consistently more even than in the non-treated, SO_2_ exposed mice (Fig. 4a). Upon imaging the alveolar region of the mouse tissue samples, it was evident that the alveolar type II cells of the AZM pre-treated mice were accumulating lamellar bodies (Fig. 4b yellow arrows). Lamellar bodies were also seen in the untreated samples, but they did not accumulate to the same degree (Fig. 4b).

## Discussion

In this study, we have administered SO_2_ gas to mice in a controlled manner to expose all test animals evenly and reproducibly. We have demonstrated that SO_2_ can be used as a challenging agent in mice to induce subtle airway epithelial damage and leakiness measured by increased epithelial shedding and blood-derived human serum albumin (HSA) extravasation into BALF. Pre-treating mice with AZM 2 weeks prior to SO_2_ exposure reduced epithelial shedding, concentrations of HSA in BALF and mitigated pro-inflammatory gene responses, demonstrating the barrier enhancing role of AZM beyond the antibiotic activity of this drug, as has been previously shown in vitro [13, 14, 16].

Air pollution from natural disasters (i.e. volcanic emissions), and those caused by human influences (i.e. forest fires) are a major concern to governments throughout the world [24]. Sulfuric oxides are continuously produced in vast quantities through the burning of fossil fuels for electricity and transportation [25, 26], as well as in sporadic disasters such as volcanic eruptions. Sulfur dioxide (SO_2_) is a major component of air pollution and is transformed into sulfuric acid in water, and when inhaled can cause damage to the respiratory system, leading to inflammation and barrier failure [27, 28]. This makes the study of SO_2_ and how it affects pulmonary function highly important and clinically relevant. With the increasing evidence for the lung protective capabilities of AZM [29–32], we decided to pre-treat mice with AZM before applying short term SO_2_ exposure and analyze the effect of AZM on prevention or attenuation of initial damage responses and the subsequent immunomodulatory effects on the epithelial barrier.

Intravenous administration of HSA into experimental animals has been widely used as a marker for vascular permeability and to study other biological problems such as drug delivery [33]. Albumin, for instance, has been used to target clarithromycin to the lung through albumin-derived microsphere carriers [33]. There has, however, been a lack of surrogate markers to measure airway epithelial barrier leakiness and in this study, we used HSA as a marker for barrier failure. By injection of HSA into the tail vein 1 h prior to BALF harvesting, we were able to measure concentrations of HSA that had leaked into BALF using ELISA. Exposing mice to SO_2_ resulted in increased concentrations of HSA in BALF which were reduced when mice were pre-treated with AZM.

We also analyzed whether native markers of oxidative stress were changed during SO_2_ exposure and performed mass spectrometric analysis of BALF which showed increased expression of several GSTs. GSTs are an integral part of the glutathione redox system, mitigating oxidative damage to tissues, including damage induced by sulfuric compounds [34]. The observation that these proteins were upregulated on SO_2_ exposure indicated that while the gross histology of the mouse lungs was unaltered, there was an active biochemical response to oxidative damage in the lungs of SO_2_ exposed mice. In addition, we were able to reduce the GST responses by pre-treating mice with AZM for 2 weeks. A reduction in the GST responses indicates that the SO_2_-induced oxidative damage was lowered in the AZM treated mice. This is in keeping with reports that AZM inhibits hypoxic lung injury in neonatal rats and ozone-induced lung inflammation in healthy human volunteers [35, 36].

SO_2_ is a well-known inducer of inflammation in the respiratory system [37]. Thus, SO_2_ exposure has been shown to increase expression of pro-inflammatory cytokines such as interleukin-6 (IL-6) and tumor necrosis factor-α (TNF-α) in mouse lungs [38]. Furthermore, nitric oxide synthase and intercellular cell adhesion molecule-1 (ICAM-1) levels have been shown to be increased in the lungs of rats following SO_2_ inhalation [39]. These data are in line with our findings that increased expression of genes positively correlated with inflammation-related pathways and were mitigated when mice were pretreated with AZM. Expression analysis of RNA from lung tissue indicates that SO_2_ exposure resulted in increased expression of inflammatory related gene sets and/or pathways. The pathway with the highest normalized enrichment score was the interferon alpha response. By focusing on the genes that were positively correlated with the interferon alpha response pathway on SO_2_ exposure, we noticed that these genes were not as highly expressed when mice had been pre-treated with AZM before SO_2_ exposure. Interestingly, expression of several additional immune-related genes followed the same trend, that is, increased expression as a result of SO_2_ challenge, but close to control expression in AZM pre-treated mice, regardless of SO_2_ exposure, as observed previously in epithelial cells stimulated with purulent exudate [40]. This indicates immunomodulatory effects of AZM, where the pre-treated mice show attenuated inflammatory responses to the SO_2_ challenge. Our results indicate that AZM should be considered as a candidate for treatment of lung injury similar to that which occurs in conditions such as acute respiratory distress syndrome (ARDS). In fact, AZM used as adjunctive therapy in patients with ARDS has been shown to be beneficial [41]. Collectively, these data demonstrate that a single exposure of SO_2_ can induce inflammation in mouse lung tissue and that this inflammatory response is dampened if mice are pretreated with AZM. As excessive inflammatory responses are hallmarks of progressive lung diseases such as ARDS, it is important to find drugs that dampen the response [42]. Further investigation into the molecular function of AZM in the airway epithelium is of great importance for several lung diseases including both life-threating conditions such as ARDS and COPD.

Using ALI cultured human bronchial epithelial cells, we recently demonstrated that AZM treatment induces epidermal differentiation, as shown by the expression of skin associated markers evaluated by gene expression analysis [16]. This study was based on the investigation of bronchial epithelial cell lines cultured in air-liquid interface conditions where AZM was added directly to the culture media, whereas the in vivo conditions we evaluated in the present study are much more complicated and represent changes following AZM administrated p.o. Nonetheless, the findings presented here show that after p.o. administration, AZM appears to protect the integrity of the respiratory epithelial barrier. In ALI cultures, AZM induces formation of multivesicular bodies and LBs [16], structures which are only found in vivo in keratinocytes generating the water barrier in skin [43]; and in the lungs, associated with club and alveolar type II cells [44]. Interestingly, an increase in the formation of LBs was confirmed in mice pre-treated with AZM, where alveolar type II cells were seen to form accumulations of LBs and in the tracheal epithelial layer, where an accumulation of vacuoles were observed. Whether formation of LBs contributes to the barrier enhancement of AE is currently not known. Pre-treated mice that had been exposed to SO_2_ showed less cellular shedding than untreated mice exposed to SO_2_, leading us to the conclusion that AZM confers barrier enhancement in the lungs.

Of interest, AZM has been previously shown to attenuate tobacco smoke-induced oxidative stress-related cell viability reduction in cultured human lung cells [45], thus supporting a protective role in an in vitro application.

An important limitation of our model is that in the current study, we only mimicked intermittent exposure of SO_2_, as opposed to prolonged exposure. As such, we represent the protective effects of AZM over a short SO_2_ exposure time period, resembling a volcanic exposure or a forest fire, but not chronic exposure like that associated with city pollution. Our data indicate however that AZM could have protective effects for individuals with weakened lung function with a foreseeable risk of exposure to SO_2_, such as during seasonal changes in city pollution or during travel. The results presented here would benefit from supporting studies using prolonged SO_2_ exposure.

## Conclusions

Collectively, we have demonstrated in this study that pre-treating mice with AZM before exposure to SO_2_ gas reduces the damage to the respiratory epithelium, including reduced epithelial leakiness and oxidative stress, and a dampening of the inflammatory response. This study and previous in vitro studies demonstrate the need for non-antibacterial drugs that maintain the disease modifying effects of AZM with less risk of causing bacterial resistance. The airway epithelium is the first line of defense against various pathogens and external hazardous particles such as SO_2_ and therefore strengthening the epithelium in patients with barrier dysfunction is of great importance, particularly for protection of immunodeficient or otherwise compromised patients from hazardous components in the air, such as pollutants and infectious agents like bacteria and viruses. Our data corroborate the notion that AZM provides this sort of protection, and that prophylactic administration of AZM to patients suffering from respiratory diseases, or preferably modified derivatives of AZM lacking antibacterial activity may be a feasible treatment option to reduce exacerbations.

## Data Availability

The datasets during and/or analysed during the current study available from the corresponding author on reasonable request.

## Abbreviations

AE: Air-Liquid Interface
Airway Epithelium: Air-Liquid Interface
ARDS: Acute Respiratory Distress Syndrome
AZM: Azithromycin
BALF: Bronchoalveolar Lavage Fluid
GST: Glutathione-S-Transferase
HSA: Human Serum Albumin
LB: Lamellar Bodies
MS: Mass Spectrometry
p.o.: per orally
SO_2_: Sulphur Dioxide
